# The Training for Masseuses

**Published:** 1916-08-05

**Authors:** Sarah Grafton

**Affiliations:** The Incorporated Society of Trained Masseuses, 157 Great Portland Street, London, W.


*To the Editor of* THE HOSPITAL.

### The Training for Masseuses.

*To the Editor of* THE HOSPITAL.

SIR,—In reference to your annotation in "Round the Hospitals" of July 29, will you kindly allow me to state on behalf of the Incorporated Society of Trained Masseuses that in regard to increasing the training in massage from six months to one year, though this question has often been discussed by members of the Society and a certain number of them are in favour of it, no pronouncement has been made by the Society that the change is to be effected, and I can safely say that we contemplate no alteration in this matter until after the war. When this subject comes up for discussion no change could of course be decided on without matrons of hospitals and trained nurses, a large number of whom belong to the Society, being first consulted and their views considered.

May I call your attention to the fact that nurses who qualify as masseuses during the period of their hospital training have always received special consideration?—Yours faithfully,

SARAH GRAFTON.

The Incorporated Society of Trained Masseuses,
157 Great Portland Street, London, W.
August 1, 1916.

